# Supplementary material for: EPISeg: Automated segmentation of the spinal cord on echo planar images using open-access multi-center data
Source: Imaging Neurosci (Camb). 2025 Sep 9;3:IMAG.a.98. doi: 10.1162/IMAG.a.98 (PMC12421696; doi:10.1162/IMAG.a.98)
Supplement: Supplementary Material [file IMAG.a.98_supp.pdf]

EPISeg: Automated segmentation of the spinal cord on echo planar images using open-access multi-center data Supplementary Material

July 1, 2025

Table 1: **Site-specific performance of EPISeg (M_f) for spinal cord segmentation.** Mean Dice Score (DS) ± standard deviation and mean 95th percentile Hausdorff Distance (HD95) ± standard deviation (in mm) for the final EPISeg (M_f) model are presented, calculated independently for each acquisition site.

Site	DS ± S.D.	HD95 ± S.D. (in mm)
Geneva_rest (N=2)	0.89 ± 0.16	1.00 ± 0.03
Hamburg_pain (N=1)	0.95 ± 0.05	1.00 ± 0.00
Zurich_Cerv_bilatmotor (N=4)	0.90 ± 0.06	1.06 ± 0.06
UNF_MSL (N=4)	0.92 ± 0.06	1.00 ± 0.03
NW_motor (N=6)	0.87 ± 0.12	1.20 ± 0.02
NW_tactile (N=6)	0.89 ± 0.09	1.13 ± 0.03
OUHSC_stim (N=6)	0.87 ± 0.08	1.29 ± 0.06
NW_motor-Weber (N=3)	0.88 ± 0.10	1.27 ± 0.02
KCL_rest (N=3)	0.84 ± 0.15	1.33 ± 0.09
Stanford_rest (N=6)	0.85 ± 0.09	1.32 ± 0.03
NW_thermal (N=3)	0.89 ± 0.05	1.13 ± 0.06
Stanford_restmartucci (N=9)	0.87 ± 0.05	1.11 ± 0.03
Zurich_Lumb_rest (N=3)	0.71 ± 0.24	3.39 ± 0.73

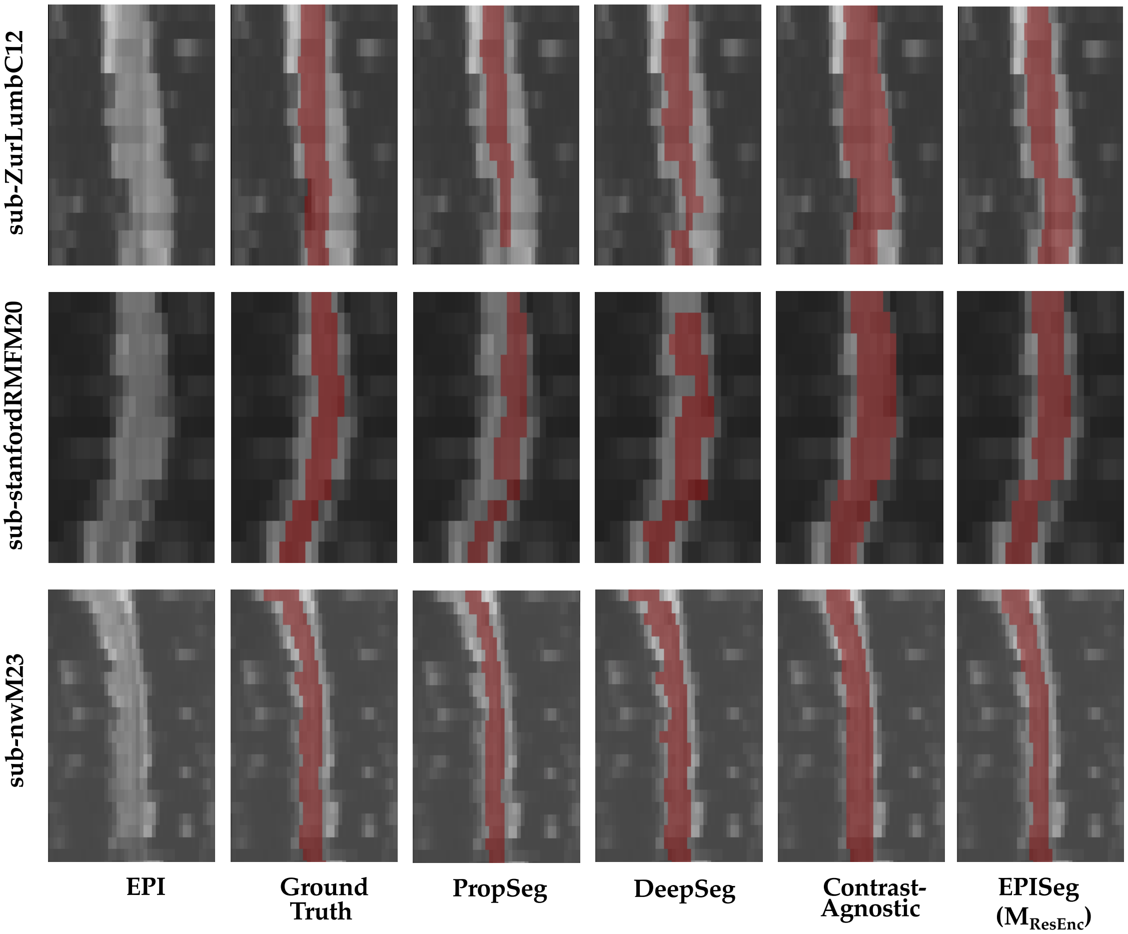

Figure 1: **Visual comparison of spinal cord segmentation performance on exemplary EPI slices.** Segmentation results for three representative subjects (sub-ZurLumbC12, sub-stanfordRMFM20, sub-nwM23) are shown. Columns display the original EPI, Ground Truth, and segmentations from PropSeg, DeepSeg, Contrast-Agnostic, and our proposed EPISeg (M_{ResEnc}) method. The red overlay indicates the segmented spinal cord. EPISeg (M_{ResEnc}) consistently yields segmentations that more closely align with the ground truth compared to other methods, demonstrating robustness across varied image characteristics.

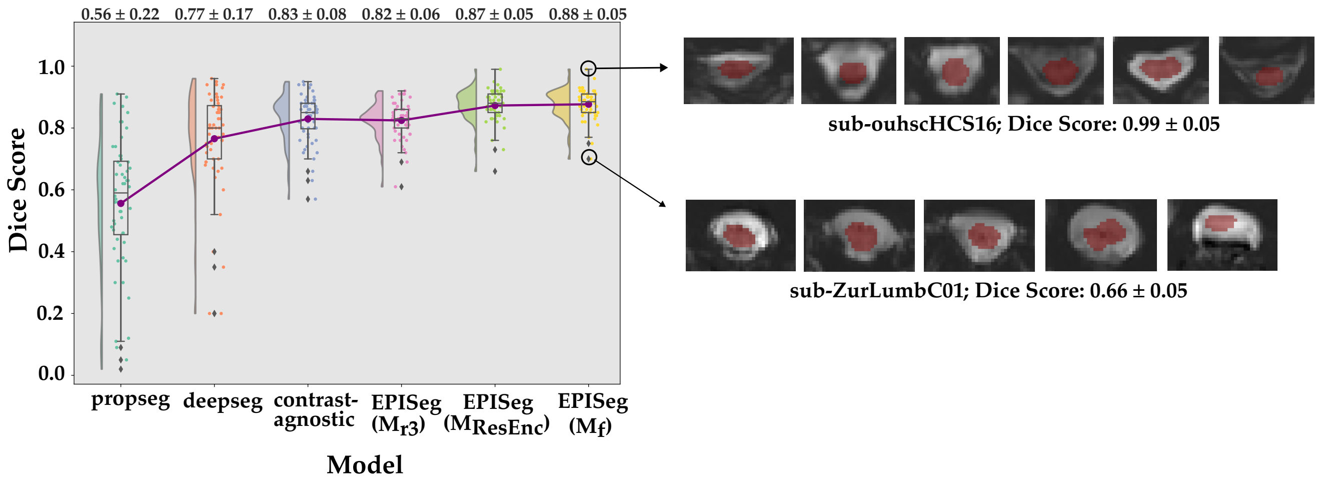

Figure 2: **EPISeg (M_f) segmentation performance range and comparative model evaluation.** Right: Examples of the best (top, subject sub-ouhscHCS16, Dice Score: 0.99 $\pm$ 0.05) and worst (bottom, subject sub-ZurLumbC01, Dice Score: 0.66 $\pm$ 0.05) spinal cord segmentations (red overlay) produced by the final EPISeg (M_f) model. Raincloud plots comparing Dice Score distributions for EPISeg (M_f) against other models: propseg, deepseg, contrast-agnostic, EPISeg (M_r3), and EPISeg (M_{ResEnc}). Mean $\pm$ standard deviation Dice Scores for each model are listed above the plots, and the purple line tracks the trend in average performance.
